# A novel index for trending sepsis and inflammatory response using processed EEG and Heart Rate Variability

**DOI:** 10.1007/s10877-026-01472-6

**Published:** 2026-07-24

**Authors:** Jaume Millán i Ichon, Montserrat Vallverdú-Ferrer, Erik Weber Jensen, Carolina Frederico Avendaño, Joy Nnagbo, Laia Yébenes-Serra, Michel M.R.F. Struys, Gertrude J. Nieuwenhuijs-Moeke

**Affiliations:** 1 Institute for Research and Innovation in Health (IRIS), School of Industrial Engineering of Barcelona (ETSEIB), Universitat Politècnica de Catalunya (UPC), CIBER of Bioengineering, Biomaterials and Nanomedicine (CIBER-BBN), Barcelona, Spain; 2 Biomedical engineering, School of Industrial Engineering of Barcelona (ETSEIB) - UPC, Barcelona, Spain; 3 Hospital Quirón Barcelona (Grupo Quirónsalud), Barcelona, Spain; 4 Anaesthesiology department, National Hospital Abuja, Abuja, Nigeria; 5 Department of Anesthesiology, University Medical Center Groningen, University of Groningen, Groningen, Netherlands; 6 Department of Basic and Applied Medical Sciences, Ghent University, Gent, Belgium; 7 Department of R&D, Coresys Health S.L, Mataró, Spain

**Keywords:** heart rate variability, electroencephalography, systemic inflammation, sepsis, adaptive neuro-fuzzy inference system, autonomic nervous system

## Abstract

Early detection of systemic inflammation and sepsis is essential for improving patient outcomes. Heart rate variability (HRV) has been proposed as a non-invasive marker of inflammation; however, its specificity is limited, particularly under anesthesia or sedation. This study introduces the Trend of Sepsis and Inflammation (TSI), a novel index combining HRV and electroencephalogram (EEG)-derived parameters to better isolate inflammation-related autonomic changes.A post-hoc observational analysis was conducted in two cohorts: surgical patients undergoing general anesthesia (*N* = 41) and septic patients admitted to the intensive care unit (*N* = 21). Continuous EEG and ECG recordings were obtained using a multimodal monitoring system. HRV parameters from time and frequency domains, together with an EEG-derived Brain Activity Index, were integrated using an Adaptive Neuro-Fuzzy Inference System (ANFIS) to compute the TSI. TSI values were compared across three conditions: awake baseline, post-surgical inflammation, and sepsis. Statistical analysis employed non-parametric tests, while discriminative performance was assessed using prediction probability (Pk) and concordance with predefined interpretation ranges.TSI values increased progressively across conditions. Median values were 2.29 (IQR: 0.00–13.74) at baseline, 24.68 (18.50–32.00) post-surgery, and 65.59 (52.38–76.25) in sepsis, with significant differences (*p* < 0.0001). The TSI demonstrated strong discriminative ability (Pk = 93.58%) and high overall concordance (92.6%). HRV spectrograms and EEG-compensated analyses supported physiological consistency.The TSI distinguishes between baseline, post-surgical inflammatory, and septic states. By compensating for anesthesia-related autonomic suppression, it provides a continuous, non-invasive measure of inflammatory burden, supporting its potential use in perioperative and critical care settings. Further validation against established biomarkers is warranted.

## Introduction

Early detection of systemic inflammation and sepsis remains a major challenge in perioperative and critical care medicine, where delayed recognition is associated with increased morbidity and mortality [1–3]. Inflammatory responses may arise from both infectious and non-infectious insults, including surgery, trauma, ischemia–reperfusion injury, and organ dysfunction, and can progress to systemic inflammatory response syndrome (SIRS) or sepsis when dysregulated [1, 4]. Despite their clinical relevance, current monitoring approaches rely largely on intermittent vital signs and laboratory biomarkers, which often lack sensitivity for early or evolving inflammatory states [5].

**Fig. 1:**
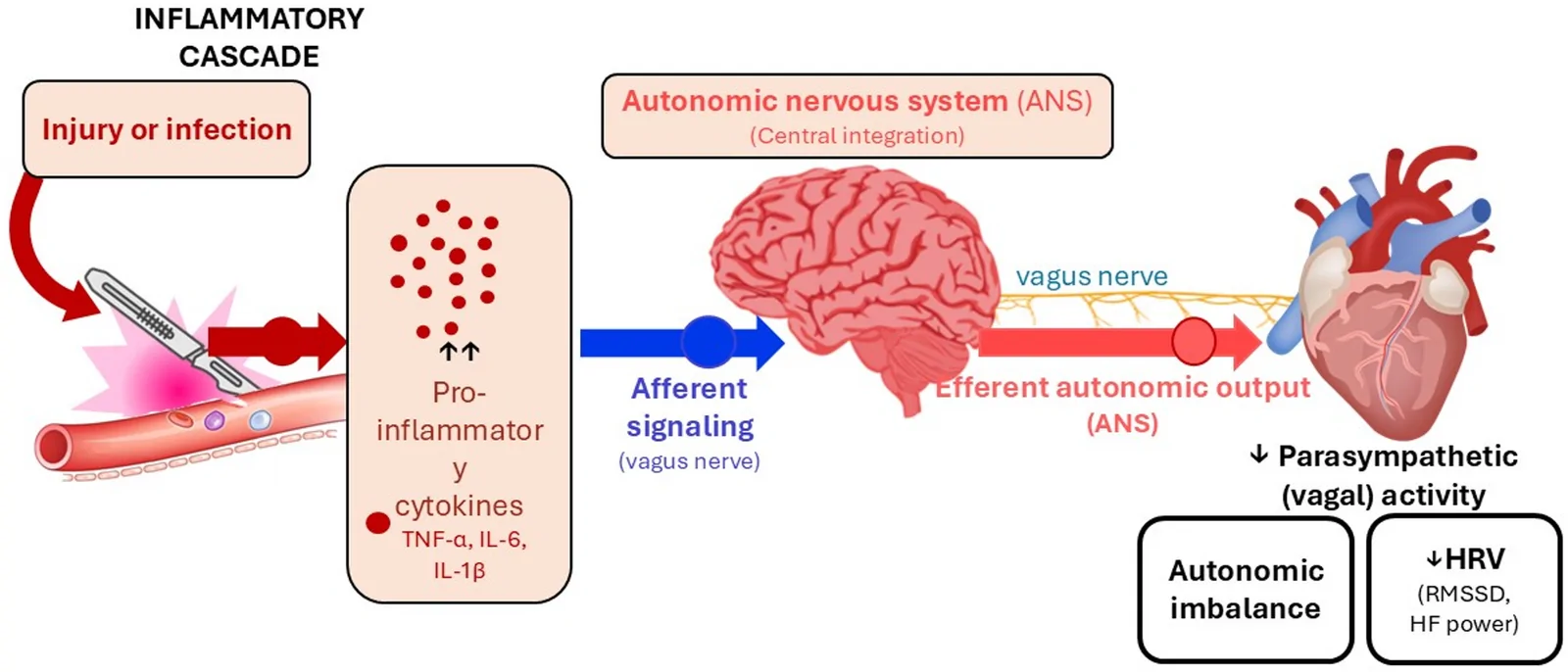
Schematic representation of the autonomic response to systemic inflammation. Tissue injury or infection induces the release of pro-inflammatory cytokines (e.g., TNF-α, IL-6, IL-1β), which signal to the central nervous system via afferent pathways, including the vagus nerve. This results in autonomic modulation, characterized by reduced parasympathetic (vagal) activity and autonomic imbalance, leading to decreased heart rate variability (HRV), reflected in measures such as RMSSD and high-frequency (HF) power

The autonomic nervous system (ANS) plays a central role in the regulation of inflammation through bidirectional communication between the immune system and the central nervous system, including vagal modulation of cytokine release [6]. This interaction provides a physiological basis for heart rate variability (HRV) as a non-invasive surrogate marker of inflammatory activity (Fig. 1). Reduced HRV, reflected in time-domain measures such as the root mean square of successive differences (RMSSD) and frequency-domain components including high-frequency (HF) and low-frequency (LF) power, has been consistently associated with systemic inflammation and sepsis [7–13]. However, HRV is highly susceptible to confounding by anesthesia, analgesia, and sedation, all of which suppress autonomic variability and limit its specificity in perioperative and intensive care settings [14–16].

**Fig. 2:**
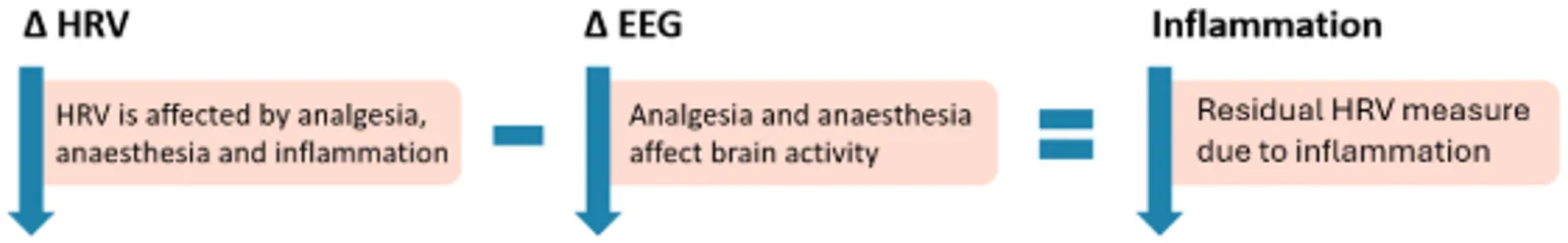
Conceptual framework for isolating inflammation-related autonomic changes. HRV variability is reduced by both systemic inflammation and pharmacological factors such as anesthesia and analgesia. EEG-derived measures primarily reflect sedation-related cortical suppression. By accounting for EEG-related effects, the residual reduction in HRV can be attributed more specifically to inflammation

To address this limitation, electroencephalography (EEG) provides an independent and well-established measure of anesthetic and sedative depth. As illustrated in Fig. 2, pharmacological suppression affects both cortical activity and autonomic output. By integrating EEG-derived metrics with HRV, it becomes possible to account for sedation-related autonomic suppression and more specifically isolate inflammation-related changes.

Adaptive Neuro-Fuzzy Inference Systems (ANFIS) provide an effective framework for this integration, combining data-driven learning with interpretable rule-based modeling to capture complex, non-linear relationships between physiological signals [17–20].

In this study, we propose the Trend of Sepsis and Inflammation (TSI v1.0a), a novel index derived from an ANFIS model integrating HRV and EEG-derived parameters. The TSI was quantified in surgical patients under general anesthesia and in septic patients in the intensive care unit. We hypothesized that incorporating EEG-derived information would mitigate sedation-related confounding of HRV, enabling improved discrimination between baseline, post-surgical inflammatory, and septic states. This work explores the feasibility of continuous, non-invasive monitoring of inflammatory burden using combined HRV–EEG analysis.

## Materials and methods

### Study Population and Eligibility Criteria

This observational study analyzed physiological data collected in two clinical cohorts: elective surgical patients at Hospital Quirónsalud, Barcelona, Spain (HQS; *n* = 41), and adult intensive care unit (ICU) patients with sepsis at National Hospital Abuja, Nigeria (NHA; *n* = 21). Both studies were approved by the respective local ethics committees (HQS protocol 2023/23-ANE-HQB; NHA protocol NHA/EC/131/2024). Written informed consent was obtained from all participants.

Eligible patients in the HQS cohort were adults undergoing elective surgery under general anesthesia with American Society of Anesthesiologists (ASA) physical status I or II. Exclusion criteria were procedures interfering with sensor placement, allergy to adhesive electrodes, neurological disorders, alcohol or substance abuse, chronic inflammatory disease, presence of a pacemaker or implantable cardioverter-defibrillator, treatment with beta-blockers or antiarrhythmic medication, patients with cardiac arrhythmia, recent cardiopulmonary arrest, and pregnancy or breastfeeding.

Eligible patients in the NHA cohort were adults admitted to the ICU with sepsis defined according to Sepsis-3 criteria [3], representing a range of clinical severities. Sepsis was either the primary diagnosis upon admission or transfer to the ICU, or it developed secondary to a surgical procedure or complication during their ICU stay. For patients who developed sepsis within the unit, clinical identification was triggered by established clinical criteria (SIRS and Sepsis-3) as part of the hospital’s standard routine. These patients were not under continuous pharmacological sedation during the monitoring sessions, the level of consciousness varied according to their severe underlying clinical condition rather than continuous drug administration. Exclusion criteria were neurological disorders, alcohol or substance abuse, chronic inflammatory disease, presence of a pacemaker or implantable cardioverter-defibrillator, patients with cardiac arrhythmia, treatment with beta-blockers, antiarrhythmic or anti-inflammatory medication, and pregnancy or breastfeeding.

The study therefore comprised two distinct clinical populations: surgical patients under general anesthesia and critically ill patients with sepsis. This design enabled comparison across three physiological states: awake baseline, surgery-associated inflammatory stress, and sepsis. All analyses were conducted post hoc, and the TSI was not used to guide clinical management.

### Electroencephalogram and Electrocardiogram Signal Recordings

Physiological signals were continuously recorded using the CoreSys One monitor (CoreSys Health SL, Barcelona, Spain), a multimodal device capable of simultaneous acquisition of two electroencephalogram (EEG) channels and one electrocardiogram (ECG) channel at a sampling frequency of 1024 Hz. All channels were time-synchronized.

**Fig. 3:**
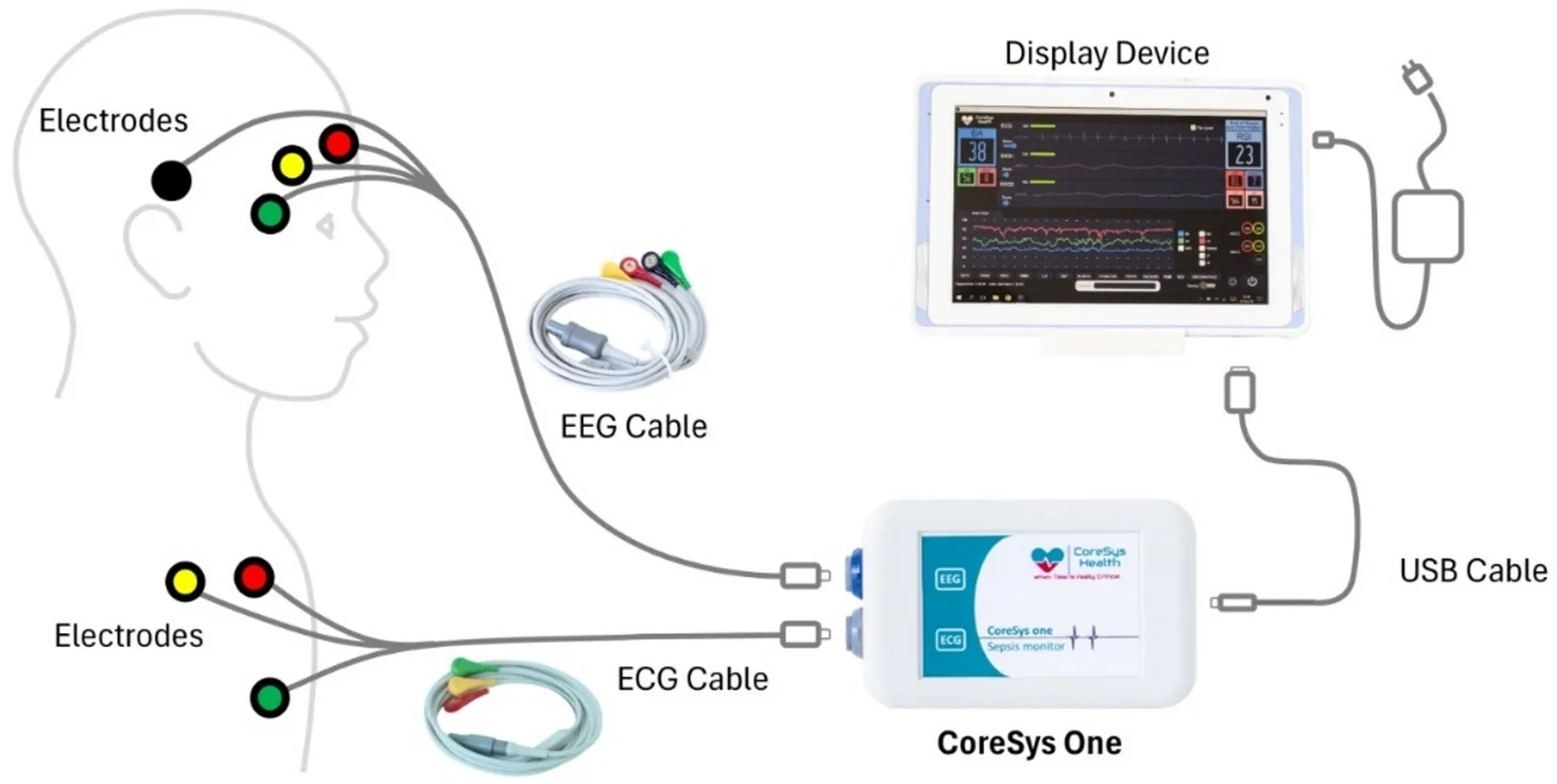
Placement of the CoreSys One device and its electrodes

EEG electrodes were positioned over the left frontal and left temporal regions, with a reference electrode placed behind the ear (Fig. 3). This configuration allowed stable signal acquisition while minimizing interference with the surgical field. In surgical patients, electrodes were removed at the end of the procedure.

Recordings in the HQS cohort ranged from 45 min to 5 h, depending on surgical duration. Recordings in the NHA cohort ranged from 1 to 4 h.

### Electrocardiogram and Electroencephalogram preprocessing

#### ECG Preprocessing and HRV Analysis

ECG signals were preprocessed to ensure adequate quality for heart rate variability (HRV) analysis. Raw ECG recordings were band-pass filtered to reduce baseline wander, high-frequency noise, and motion artifacts. R-wave detection was performed using the Pan-Tompkins algorithm [21], which identifies QRS complexes through adaptive thresholding of the differentiated and integrated ECG signal. Detected R-R intervals were subsequently screened for signal quality, and ectopic beats and artifacts were excluded to avoid distortion of HRV metrics.

HRV analysis was performed using sliding windows of 180 s with 30 s overlap, balancing temporal resolution against the stability of time- and frequency-domain estimates. Within each window, HRV parameters were calculated according to established recommendations [22].

Time-domain analysis included the root mean square of successive differences (RMSSD), reflecting short-term variability predominantly mediated by parasympathetic activity. Mean heart rate (HR) was calculated as the inverse of the mean R-R interval and expressed in beats per minute.

Frequency-domain analysis included estimation of low-frequency (LF; 0.04–0.15 Hz) and high-frequency (HF; 0.15–0.40 Hz) spectral power. LF power reflects mixed autonomic influences, whereas HF power is primarily associated with parasympathetic modulation. The LF/HF ratio was also calculated as a composite frequency-domain feature. In addition, time-frequency HRV spectrograms were generated to visualize the temporal evolution of spectral energy under baseline, post-surgical, and septic conditions. The HRV-derived features used as inputs to the TSI model were HR, RMSSD, and LF/HF.

**Fig. 4:**
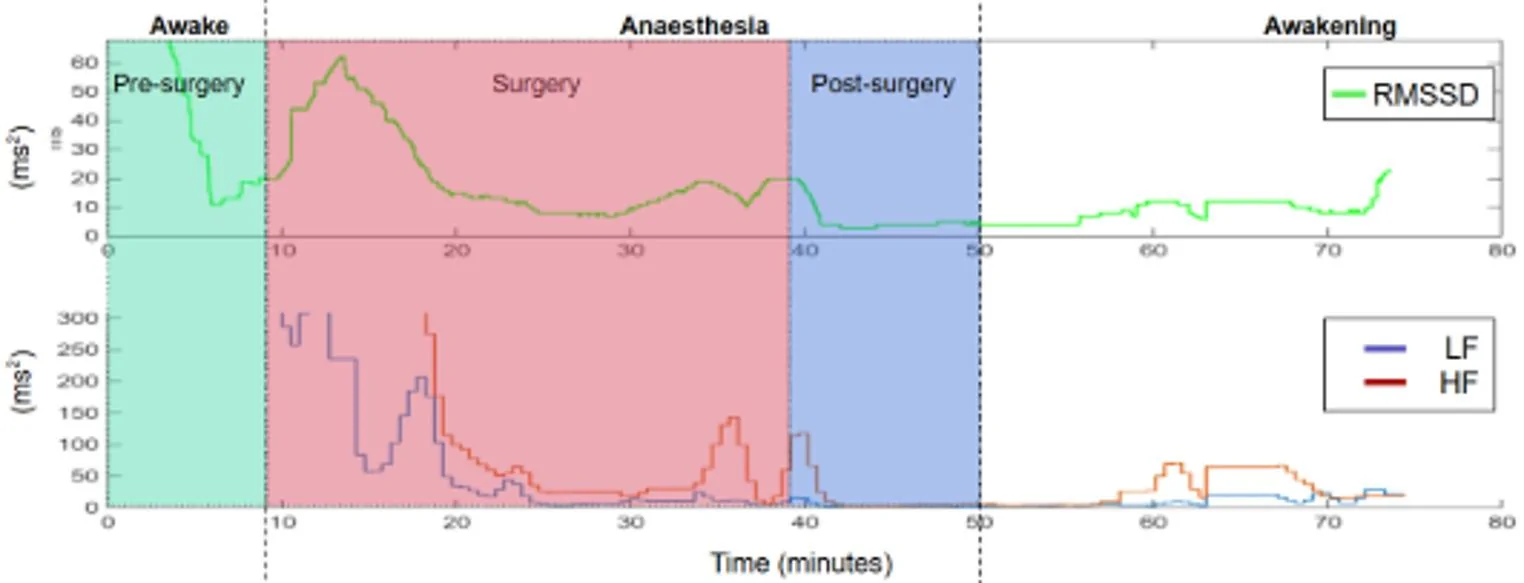
An example of a HRV recording from a surgical patient. The upper panel shows the RMSSD and the bottom the low (LF) and high frequency (HF) components. The green and blue segments were used for the analysis

A representative HRV recording from a surgical patient is shown in Fig. 4.

#### EEG Preprocessing and Analysis

EEG signals were band-pass filtered between 0.5 and 44 Hz to reduce baseline drift, electromyographic contamination, and high-frequency noise. Notch filtering at the local power-line frequency was applied to suppress electrical interference and harmonics. Frequency-domain analysis was performed using the Fast Fourier Transform (FFT) [23] to compute absolute and normalized spectral power[24].

These EEG spectral features were processed using an Adaptive Neuro-Fuzzy Inference System (ANFIS) to derive the Brain Activity (BA v1.0a) index. The BA model used four spectral inputs defined as logarithmic power ratios: delta-band power (0.5–3 Hz) relative to broadband power (0.5–30 Hz), beta-band power (16–30 Hz) relative to broadband power, high-frequency activity (30–44 Hz) relative to mid-frequency activity (11–22 Hz), and alpha-band power (8–13 Hz) relative to broadband power. In addition, burst suppression (BS) ratio was included to detect deep anesthesia.

The BA index was developed using the Bispectral Index (BIS) as the output reference during model training. Specifically, data from 20 patients in the HQS cohort were used to train the BA model to track BIS behavior. As a result, the BA index was scaled analogously to BIS, with higher values (80–100) corresponding to wakefulness and progressively lower values reflecting deeper sedation or anesthesia. Values of approximately 80–70 indicate mild sedation, 60–70 deep sedation, and 40–60 general anesthesia. BA values below 40 are associated with EEG suppression patterns, and a value of 0 corresponds to an isoelectric EEG. In the present study, BA was used as an EEG-derived measure of hypnotic or sedative state to account for pharmacological suppression when interpreting HRV-derived parameters.

### Signal Selection and Dataset Construction

This study evaluated the Trend of Sepsis and Inflammation (TSI) under three physiological conditions: awake baseline, post-surgical inflammatory stress, and sepsis.

For the HQS cohort, two 10-min signal segments were extracted from each patient for both EEG and ECG analysis. The baseline segment corresponded to the 10-min period before loss of consciousness, representing the awake and non-inflamed condition. The post-surgical segment corresponded to the 10-min period immediately preceding recovery of consciousness, representing the late intraoperative period and expected surgery-associated inflammatory stress.

For the NHA cohort, one continuous 10-min artifact-free segment was selected from each of the 21 ICU patients with sepsis. For the majority of the patients, the physiological recordings of EEG and HRV were performed upon admission to the ICU. For the subset of patients who developed sepsis during their ICU stay, the 10-min recordings were obtained immediately following the formal clinical confirmation of sepsis. These segments were chosen to represent the active septic state during stable recording periods.

Given the limited cohort size, validation was performed at the segment level; future studies should assess subject-level generalization. To construct training and validation datasets, temporally distinct and non-overlapping signal segments were used. In the HQS cohort, validation segments were selected from periods separate from those used for training; for the post-surgical condition, validation segments consisted of 10-min intervals immediately preceding the training segments. In the NHA cohort, validation segments were selected from recording periods distinct from those used during model development. Although some subjects contributed data to both model development and validation, no segment used for parameter estimation was reused for performance evaluation. Accordingly, validation was performed at the segment level rather than as a fully independent subject-level validation.

### TSI index description

The Trend of Sepsis and Inflammation (TSI) was derived using an Adaptive Neuro-Fuzzy Inference System (ANFIS) implemented in MATLAB (MathWorks, Natick, MA, USA). ANFIS combines fuzzy logic inference with data-driven learning to model nonlinear relationships between physiological inputs and a continuous output. Model training was performed using a hybrid learning algorithm combining least-squares estimation and backpropagation-based gradient descent over 400 epochs.

The model used four input variables: HR, RMSSD, LF/HF, and the EEG-derived BA index. These inputs were selected to capture complementary information on autonomic regulation and cerebral state. The ANFIS architecture employed two Gaussian membership functions per input and a constant output membership function.

A total of 30 patients were used for TSI model training, including 20 patients from the HQS cohort and 10 septic patients from the NHA cohort. The training set comprised three physiological groups: baseline, post-surgical, and septic states. These groups were assigned reference target values of 0, 30, and 100, respectively, defining the intended operating scale of the TSI. These values were selected to represent relative operating ranges and were not intended as calibrated biological thresholds. Approximately 1,800 samples were extracted from the selected segments for model training.

To reduce the risk of overfitting, the ANFIS model was intentionally kept compact. With four inputs and two membership functions per input, the total number of model parameters was 96, yielding an observation-to-parameter ratio of approximately 18:1. Training and validation used temporally distinct, non-overlapping segments, thereby limiting information leakage between model development and evaluation.

**Fig. 5:**
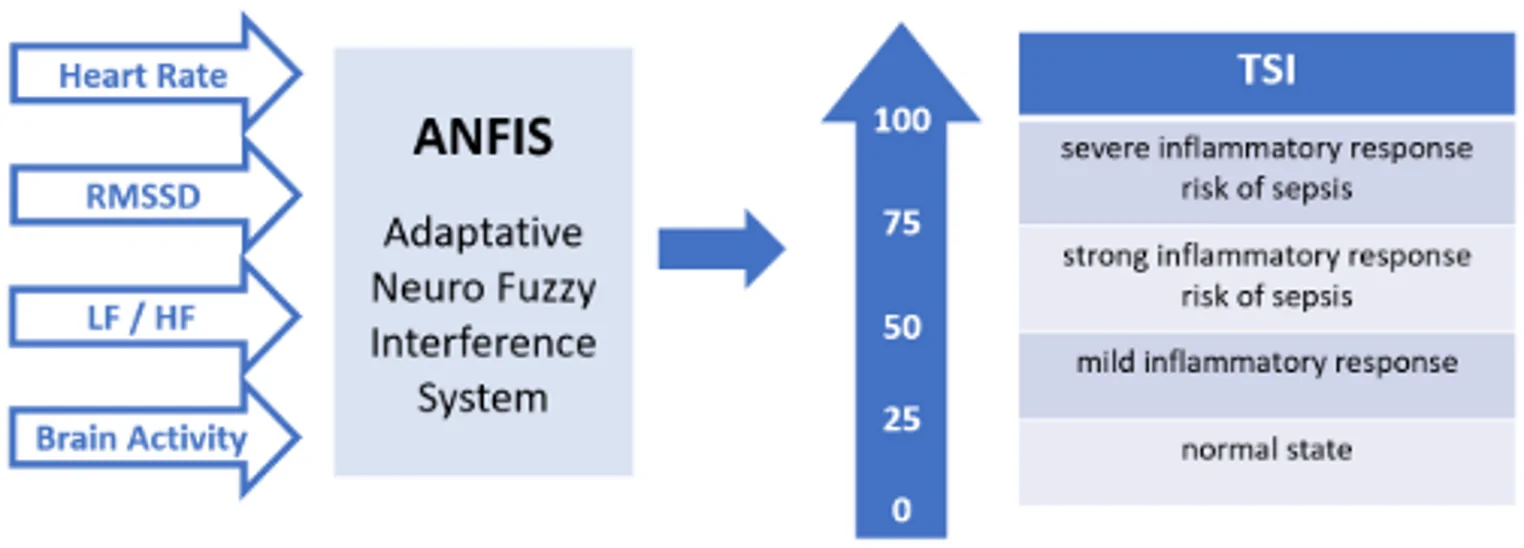
Schematic representation of the ANFIS-based TSI model implemented in the CoreSys One system. Model inputs include heart rate (HR), root mean square of successive differences (RMSSD), low-frequency to high-frequency power ratio (LF/HF), and the EEG-derived Brain Activity (BA) index

The ANFIS integrates the four physiological inputs into a single continuous output, the TSI, scaled from 0 to 100. Lower values correspond to preserved autonomic variability and minimal inflammatory burden, whereas higher values reflect progressive physiological alterations associated with increasing inflammatory stress and clinically established sepsis. The TSI was therefore designed as a continuous trend index rather than a binary diagnostic classifier. A schematic representation of the ANFIS-based TSI model is shown in Fig. 5.

### Statistical and Predictive Analysis

Statistical analyses were performed to assess the ability of the TSI to discriminate between baseline, post-surgical, and septic conditions. Because normality assumptions were not met, non-parametric methods were used throughout.

Statistical analyses were performed on patient-level median TSI values. Within the surgical cohort, paired comparisons between baseline and post-surgical TSI values were performed using the Wilcoxon signed-rank test. Comparisons between the surgical and septic cohorts, which represented independent samples, were performed using the Wilcoxon rank-sum test (Mann-Whitney U test). All tests were two-tailed, and statistical significance was defined as *p* < 0.05.

Discriminative performance was further quantified using prediction probability (Pk), which estimates the probability that the TSI correctly ranks two observations from different physiological states. Pk ranges from 0.5, indicating no discriminative ability, to 1.0, indicating perfect discrimination.

As an exploratory assessment of practical interpretability, TSI values were also evaluated relative to predefined operating ranges. Baseline segments were considered concordant when TSI was < 25, post-surgical segments when TSI was < 50, and septic segments when TSI was > = 50. Range-based concordance was defined as the proportion of segments within each group whose TSI values fell within the expected range. Overall concordance was calculated across all analyzed segments.

Together, non-parametric testing, prediction probability analysis, and exploratory range-based concordance were used to evaluate the statistical significance, discriminative performance, and practical interpretability of the TSI across the three physiological conditions.

## Results

### Study population and baseline characteristics

The study included 62 patients: 41 surgical patients in the HQS cohort and 21 septic patients in the NHA cohort. Demographic characteristics of both cohorts are summarized in Table 1. The mean age was 54.1 ± 14.8 years in the surgical cohort and 52.3 ± 15.2 years in the septic cohort, with a female proportion of 61% and 47.6%, respectively. The septic cohort comprised patients with heterogeneous etiologies, including urosepsis, postoperative sepsis, and neurosepsis.

**Table 1:** Demographic characteristics of the surgical (HQS) and septic (NHA) patient cohorts

Cohort	*N*	Mean age ± SD (yr)	Female (%)
Surgical (HQS)	41	54.1 ± 14.8	61
Septic (NHA)	21	52.3 ± 15.2	47.6
Total	62	53.4 ± 15.0	54.8

*N: number of patients; HQS: Hospital quiron Salud; NHA: National Hospital Abuja;

Details of surgical procedures within the HQS cohort are provided in Table 2 in the supplemental material. All surgical patients received total intravenous anesthesia with propofol and remifentanil.

A detailed overview of individual clinical characteristics in the septic cohort is provided in Table 3 in the supplemental material.

During model development, performance on the training dataset showed a prediction probability (Pk) of 0.956 (SE 0.0011) and a Pearson correlation coefficient (R²) of 0.897 between predicted and reference values. Performance on the validation dataset remained high, with a Pk of 0.936.

### Autonomic and Electrophysiological Patterns Across Inflammatory States

**Fig. 6:**
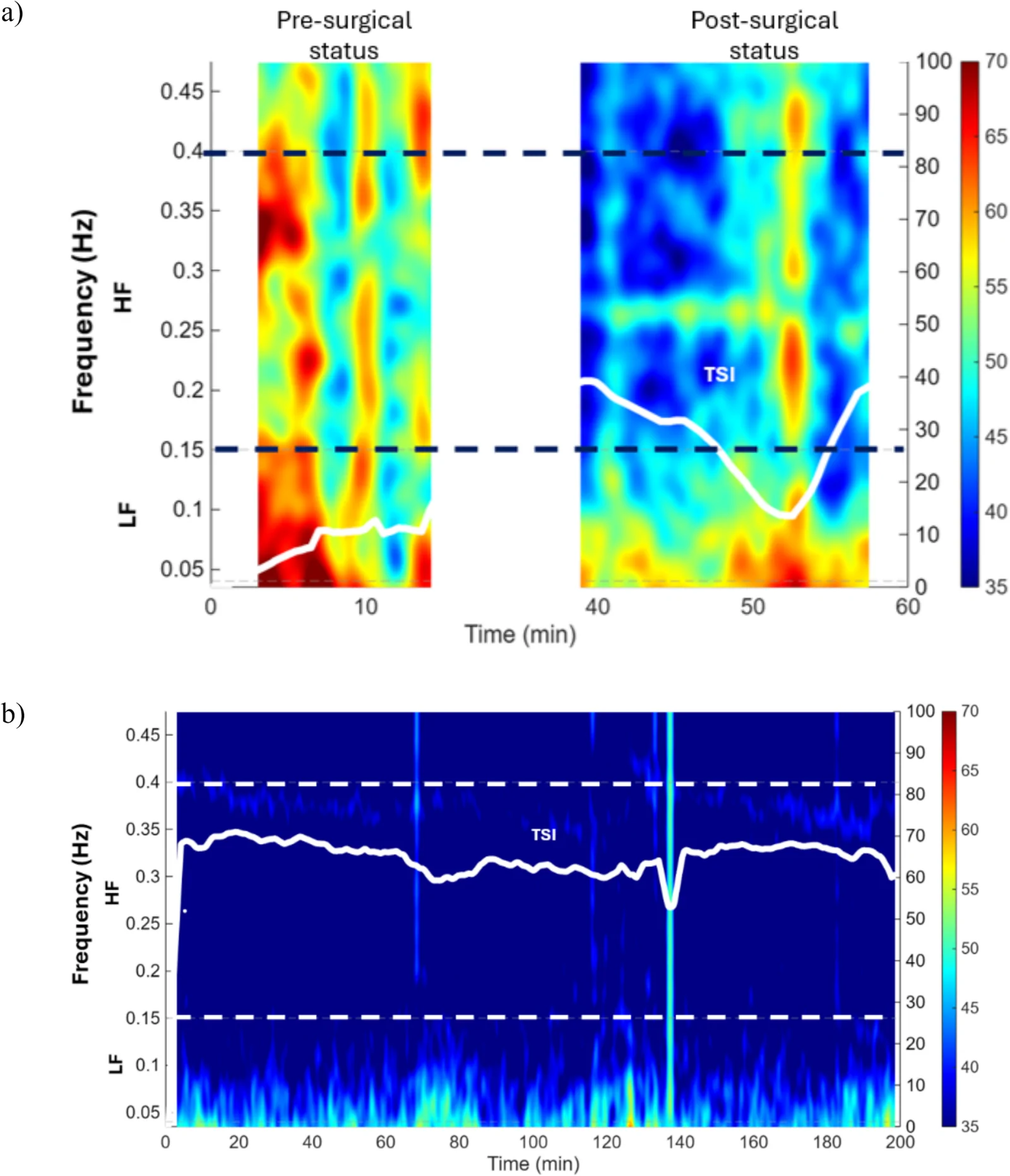
Representative HRV spectrograms showing autonomic frequency modulation in relation to inflammation. (a) Comparison between pre-surgical and post-surgical HRV spectrograms from a single patient, where a reduction in LF and HF power is observed following surgical stimulus. The white trace indicates the corresponding Trend of Sepsis and Inflammation (TSI) index (b) HRV spectrogram from a septic patient, showing decreased spectral energy in both low-frequency (LF) and high-frequency (HF) bands. The colormap scale is in dB/Hz

Figure 6 presents representative HRV spectrograms illustrating frequency-domain autonomic dynamics across increasing inflammatory burden. These visualizations provide qualitative context for the TSI by showing progressive attenuation of HRV spectral power from baseline to post-surgical and septic conditions.

Figure 6a shows pre-surgical and post-surgical HRV spectrograms from the same patient in the HQS cohort. Following surgery, a reduction in spectral power is observed in both the LF and HF bands, consistent with reduced autonomic variability associated with surgical stress and the post-surgical inflammatory state. Figure 6b shows an HRV spectrogram from a patient in the ICU cohort with clinically diagnosed sepsis, demonstrating a marked reduction in spectral energy across both LF and HF bands, consistent with severe autonomic dysregulation.

### TSI Distribution Across Baseline, Post-Surgical, and High Inflammatory States

**Fig. 7:**
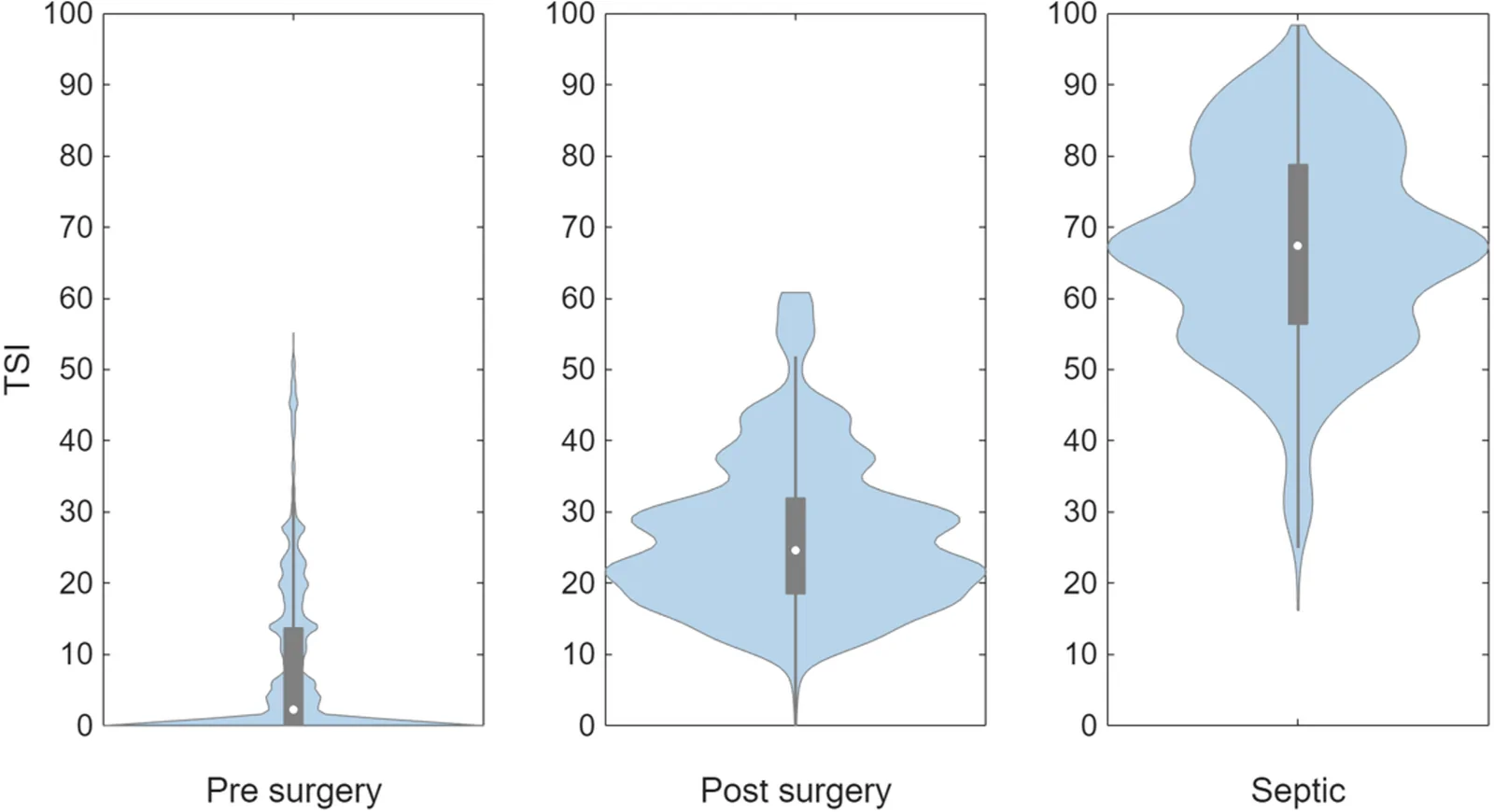
Distribution of the Trend of Sepsis and Inflammation (TSI) index across pre-surgical, post-surgical, and septic conditions. Violin plots represent the probability density of the TSI values for each group. The central box indicates the interquartile range the white dot represents the mean. The TSI increased progressively from pre-surgical to post-surgical and septic states, reflecting the expected escalation of systemic inflammation

Across the full cohort, TSI values increased progressively with increasing inflammatory severity (Fig. 7). Median TSI values were 2.29 (interquartile range, IQR: 0.00-13.74) during the awake baseline state, 24.68 (IQR: 18.50–32.00) during the post-surgical state, and 65.59 (IQR: 52.38–76.25) in the septic ICU cohort.

Within the surgical cohort, paired comparison of baseline and post-surgical values using the Wilcoxon signed-rank test demonstrated a significant increase in TSI (*p* < 0.0001). Comparisons between the post-surgical group and the septic ICU cohort using the Wilcoxon rank-sum test also showed significant differences (*p* < 0.0001). These findings indicate that the TSI discriminates between low, intermediate, and high inflammatory burden. Prediction probability across the three states was 0.9358, indicating strong threshold-independent discriminative performance.

### Exploratory Severity-Based Interpretation of the TSI Index

As an exploratory assessment of interpretability, TSI values were evaluated relative to predefined operating ranges. Baseline segments were considered concordant when TSI was < 25, post-surgical segments when TSI was < 50, and septic segments when TSI was > = 50. Using these ranges, concordance was highest in the post-surgical group (95.9%), followed by the baseline group (91.7%) and the septic cohort (90.3%), resulting in an overall concordance of 92.6%.

These ranges reflect intended levels of inflammatory burden rather than validated diagnostic thresholds. Accordingly, elevated TSI values should be interpreted as indicating increased inflammatory stress, irrespective of infectious or non-infectious etiology.

## Discussion

Heart rate variability (HRV) has been widely proposed as a non-invasive marker of systemic inflammation; however, its clinical adoption has been limited by poor specificity. HRV is influenced by numerous physiological and pharmacological factors, including anesthesia, analgesia, sedation, and stress, complicating its interpretation in perioperative and critical care settings. The present study addresses this limitation by integrating electroencephalography (EEG) with HRV to contextualize autonomic suppression and improve sensitivity to inflammation-related physiological changes. The primary clinical utility of incorporating the EEG-derived Brain Activity (BA) index is to account for the known effects of anesthetics and sedatives on heart rate variability (HRV). Because both anesthetic agents and systemic inflammation may suppress autonomic activity, an objective measure of cerebral state may provide important physiological context when interpreting HRV-derived parameters. The BA index was therefore incorporated as a surrogate measure of hypnotic state within the multimodal TSI framework.

Importantly, EEG changes are not specific to anesthesia or sedation. Accumulating evidence suggests that systemic inflammation and sepsis may themselves alter cortical activity through neuroinflammatory and metabolic mechanisms [25–29]. Therefore, the observed EEG parameters reflect a mixture of multiple sources of alterations in cerebral activity, as both drug-induced sedation and pathophysiological inflammatory processes (such as sepsis-associated encephalopathy) can independently change levels of consciousness and shift spectral properties in overlapping directions. Consequently, the BA index captures the net effect of these interacting processes and cannot currently be considered an isolated marker of either sedation or inflammation per se. Instead, within the multimodal TSI framework, it serves as a complementary, objective physiological descriptor that provides crucial context to help account for and compensate for the known suppressive effects of anesthetics and sedatives on HRV parameters.

Beyond its role as a general measure of cerebral state, previous experimental models and clinical critical care studies highlight how central neuroinflammatory dysfunction alters electrofisiological pathways. For example, White et al. [25] reported persistent delta activity and slow-wave increments in animal models of inflammation, while Gjini et al. [26] demonstrated that reduced cortical connectivity correlated with inflammation and delirium severity. In the clinical ICU setting, sepsis-associated encephalopathy is frequently accompanied by distinct EEG abnormalities, including increased theta and delta activity, triphasic waves, burst suppression, and periodic epileptiform discharges [27, 28, 29]. Together, these observations confirm that EEG dynamics capture aspects of neurological status that overlap with pharmacological effects. Combining these metrics with HRV features enhances overall sensitivity to the systemic inflammatory burden, though future prospective designs will be required to mathematically disentangle the exact proportions of this electrophysiological mixture.

Current clinical assessment of systemic inflammation relies predominantly on circulating biomarkers obtained through blood sampling. While informative, these measurements are invasive, intermittent, and subject to processing delays, limiting their utility for continuous monitoring. In contrast, the TSI is designed as a non-invasive, real-time index derived from routinely available physiological signals. Whereas HRV and processed EEG have traditionally been used to assess autonomic regulation, hypnotic state, or nociception [30], the TSI represents a shift toward integrated neurocardiac monitoring of inflammatory dynamics. The present results demonstrate that this combined approach can discriminate between baseline physiology, surgery-associated inflammation, and high inflammatory burden, supporting its potential utility as a complementary monitoring tool in perioperative and critical care environments.

Several limitations should be considered. First, no inflammatory biomarkers were available as an external reference standard, precluding definitive conclusions regarding the specificity of the TSI. Surgical trauma and clinically diagnosed sepsis were therefore used as proxy conditions associated with increasing inflammatory burden. Accordingly, the TSI should be interpreted as a physiological trend index rather than a diagnostic biomarker, and the findings should be considered exploratory.

Second, the reference ranges used to define TSI operating states were selected to reflect intended physiological conditions rather than calibrated biological thresholds. Although discrimination performance was assessed using threshold-independent metrics, these choices may influence range-based concordance and require refinement in future work.

Third, the septic cohort was relatively small and heterogeneous in etiology and severity, which may limit generalizability. In addition, although training and validation were performed on non-overlapping signal segments, some subjects contributed data to both phases. Therefore, the results reflect segment-level validation and should not be interpreted as full subject-level generalization.

Furthermore, important potential confounders including mechanical ventilation settings, vasopressor use, fluid administration, and standardized illness severity scores (e.g. SOFA and APACHE) were not systematically collected and could therefore not be incorporated into the present analyses.

Despite these limitations, the principal strength of the TSI lies in the integration of EEG and HRV. While HRV alone is sensitive but non-specific, EEG provides contextual information on cerebral and pharmacological influences, enabling more specific interpretation of autonomic changes.

Future prospective studies incorporating longitudinal measurements and established inflammatory biomarkers will be essential to validate the clinical relevance of the TSI. In particular, evaluating TSI dynamics over time and in response to therapeutic interventions may clarify its role in clinical decision-making.

In summary, the TSI shows promise as a non-invasive, continuous monitoring tool for systemic inflammatory burden. With further validation, it may complement existing biomarker-based approaches by enabling earlier detection and longitudinal tracking of inflammatory dynamics in perioperative and critical care settings.

**Table 2:** Demographic information of the patients from the HQS cohort according to surgical procedure including gender (Female or Male), age and BMI

Surgery	Patients	Gender (M/F)	Age (yr)	BMI (kg/m²)	ASA (I/II)
Abdominoplasty	1	0/1	40	21	1/0
Bilateral inguinal hernia repair	2	2/0	55.5 (55–56)	27.5 (26–29)	1/1
Cholecystectomy	6	2/4	53.8 (44–76)	26.3 (24–29)	0/6
Epigastric hernioplasty	2	2/0	68 (52–84)	31.5 (30–33)	0/2
Hemorrhoidectomy	2	0/2	58 (55–61)	24.5 (21–28)	0/2
Ileostomy closure	1	1/0	41	25	0/1
Incisional hernia repair	2	1/1	67.5 (60–75)	29.5 (29–30)	0/2
Inguinal hernia repair	2	2/0	56 (50–62)	22.5 (22–23)	1/1
Laparoscopic bilateral Adnexectomy	1	0/1	49	30	0/1
Laparoscopic bilateral inguinal hernia repair	2	2/0	54.5 (49–60)	23.5 (23–24)	0/2
Laparoscopic cholecystectomy	3	0/3	50.3 (43–63)	22 (21–23)	1/2
Laparoscopic incisional hernia repair	3	3/0	57 (54–61)	26 (22–28)	0/3
Laparoscopic inguinal hernia repair	5	3/2	56.4 (43–77)	25.2 (23–26)	1/4
Laparoscopic right adnexectomy	1	0/1	44	29	0/1
Mastopexy with implants	2	0/2	28.5 (20–37)	21.5 (20–23)	1/1
Parotidectomy	1	0/1	48	27	0/1
Percutaneous lumbar spinal fusion	1	1/0	59	26	1/0
Reservoir placement	1	0/1	59	32	0/1
Retroperitoneal tumor Resection	1	1/0	69	27	0/1
Spigelian and abdominal Hernia repair	1	0/1	72	23	0/1
Umbilical hernia repair	2	2/0	51 (48–54)	24.5 (22–27)	0/2

*Age in years; F: Female; M: Male;

**Table 3:** Demographic information of the patients from the ICU NHA cohort database

Clinical state	Age	Gender
Acute kidney injury (a sudden decline in kidney function) requiring dialysis	61	F
Acute kidney injury associated with HELLP syndrome	35	F
Addisonian crisis with sepsis following transsphenoidal pituitary adenoma removal	64	M
Hypertensive encephalopathy with sepsis of unknown origin	41	F
Ischaemic stroke with sepsis of undetermined source	62	M
Multiple cerebral infarcts with sepsis, aspiration pneumonitis and diabetes	72	F
Neurosepsis following craniotomy	67	M
Neurosepsis with concurrent anaemia	60	M
Pericardial effusion and heart failure; recent febrile episodes suggestive of infection	28	M
Persistent sepsis three days post-craniotomy	52	F
Polytrauma with diaphragmatic hernia	23	F
Puerperal sepsis with pulmonary oedema	42	F
Renal impairment due to sepsis following partial gastrectomy	40	M
Sepsis due to ruptured appendix	73	M
Sepsis secondary to infected bladder	64	F
Sepsis suspected to originate from gangrenous foot or pulmonary infection	55	M
Severe urosepsis and ARDS; CT showed multiple cerebellar infarcts	62	M
Trauma-related sepsis	30	M
Urosepsis	61	F
Urosepsis following prostatectomy; complicated by bicytopenia and acute kidney injury (a sudden decline in kidney function)	62	M

*Age in years; F: Female; M: Male;

## Data Availability

No datasets were generated or analysed during the current study.
